# A New Approach to Cultivating Growth Mindset: Strengths-Based Training for First-Year Medical Students

**DOI:** 10.1007/s40670-026-02786-5

**Published:** 2026-06-19

**Authors:** Sarah Araten, Shivani Gokhale, Archana Pradhan, Ralph A. Gigliotti, Betsy Mathew

**Affiliations:** 1 Rutgers Robert Wood Johnson Medical School, New Brunswick, NJ USA; 2 Rutgers Robert Wood Johnson Medical School, Department of Obstetrics, Gynecology and Reproductive Sciences, General Division, New Brunswick, NJ USA; 3 Office of the President, Rutgers University, New Brunswick NJ, USA; 4 Department of Family Medicine and Community Health, Rutgers Robert Wood Johnson Medical School, New Brunswick, NJ USA

**Keywords:** Strengths, Growth mindset, Medical education, Preclinical, Student development

## Abstract

Medical students face challenges that growth mindset may help address. We introduced a curriculum to help students recognize personal strengths and learn growth mindset. Quantitative (*n* = 663) and qualitative (*n* = 169) evaluations showed strong receptivity and frequent endorsement of key growth mindset principles, supporting the value of this intervention.

**Supplementary Information:**

The online version contains supplementary material available at https://doi.org/10.1007/s40670-026-02786-5.

Medical students face challenges including imposter syndrome, academic pressure from examinations, and steep learning curves. Providing medical students with a research-informed framework to address these challenges is critical [1]. One framework involves the cultivation of a growth mindset, which can be achieved by mastering seven tenets: 1. Believing effort yields improvement, 2. Embracing challenges, 3. Learning from criticism, 4. Persisting through setbacks, 5. Prioritizing learning over approval, 6. Viewing others’ success as inspiration, and 7. Believing in the malleability of intelligence [2]. Strengths-based training can support professional development for healthcare students [3] and may shift student thinking from a deficit-based framework toward a positive growth-oriented approach [4], which may also combat feelings of imposter syndrome prevalent in medical students. *CliftonStrengths*^®^ is a tool that helps learners discover their unique strengths and has established use in higher education and healthcare [3]. *CliftonStrengths*^®^ focuses on identifying talents that can be intentionally developed over time, aligning with growth mindset principles [3].

We created required activities early in the first year to guide students in recognizing their strengths and cultivating a growth-oriented approach to their education (Fig. 1).

**Fig. 1:**
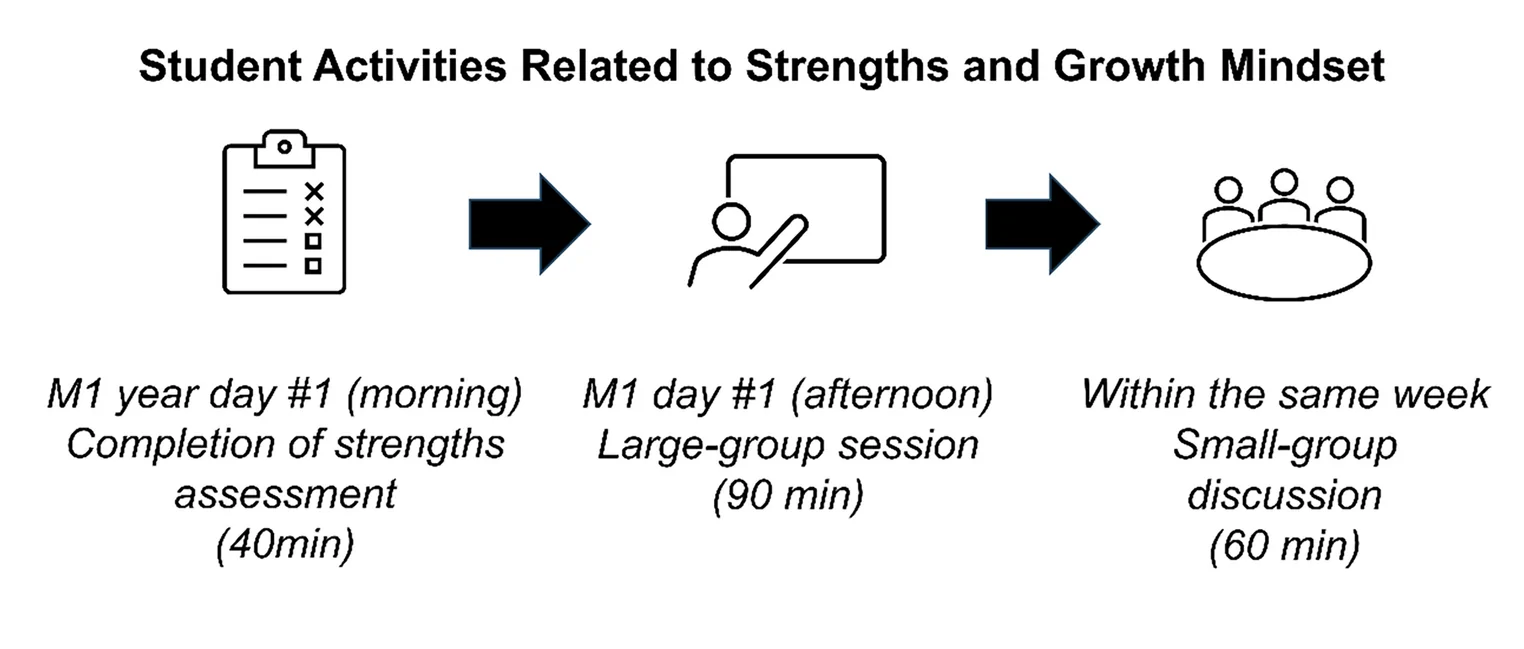
Student Activities Related to Strengths and Growth Mindset

Starting in 2020, all first-year students completed the *CliftonStrengths*^®^ *for Students* assessment, received a report of their top five strengths, attended a large-group session focused on the intersection of strengths and growth mindset, and discussed their strengths in small groups led by physician facilitators. The program objectives included the following: 1. Describe core concepts associated with leadership and communication connections, strengths-based leadership, and leading with a growth mindset. 2. Identify strengths using the *CliftonStrengths*^®^ assessment. 3. Identify ways to leverage strengths to overcome challenges, build resiliency, and reach your personal, academic, and career goals. 4. Explore strategies for cultivating, supporting, and encouraging growth mindset and strengths-based leadership. 5. Build the small-group learning community by discussing the distribution of strengths in small-group teams and how the distribution of strengths may influence group and learning dynamics.

Data was collected from anonymous student evaluations of the program from 2020 to 2024 (*n* = 663). Students evaluated the large-group session on a Likert scale (strongly agree to strongly disagree) using the question “This session was effective in meeting the learning objectives.” In 2024, students (*n* = 169) were also asked the open-ended question: “How might you consider applying what you learned from this session to your experience as a first-year student?” Responses to this question were qualitatively analyzed to evaluate familiarity with growth mindset tenets. Responses were codified by two coders using deductive thematic analysis based on the seven growth mindset tenets. Comments could be coded to multiple tenets if appropriate, and the number of comments referencing each growth mindset tenet was tallied.

Likert data demonstrated that 89% of students strongly agreed (412/663) or agreed (181/663) that the large-group session was effective in meeting learning objectives. On qualitative analysis, the growth mindset tenets that were mentioned most were Tenet 1: “Believing effort yields improvement” and Tenet 4: “Persisting through setbacks” (Table 1).

**Table 1:** Analysis of Student Responses

Analysis of Student Responses to Open-Ended Question (2024, *N* = 169)		
63/169 (37%) of responses showed familiarity with growth mindset tenet(s) in response to the following question: *How might you consider applying what you learned from this session to your experience as a first-year student?*		
Tenet	# of Responses	Student Quote Expressing Tenet
Tenet 1 – Believing effort yields improvement	33	“I cannot expect to nail everything, but I can keep practicing and working hard to improve. I have the ability to keep growing as a student and excelling.”
Tenet 2 – Embracing Challenges	7	“This lecture has definitely been something I can use to stay grounded during times where I feel scared of making mistakes. The idea of a growth mindset will hopefully remind me to not shy away from challenges but instead see them as ways to make me an even better future physician.”
Tenet 3 – Learning from Criticism	7	“Being receptive to feedback and listening to others in order to formulate new ideas were two huge takeaways from this lecture that I would like to apply in this coming year.”
Tenet 4 – Persisting Through Setbacks	28	“Going forward, if I fail at something or do not achieve a goal, I am going to reflect on it and try to understand how I can learn and grow from the experience.”
Tenet 5 – Prioritizing Learning over Approval	2	“I will try to maintain a growth mindset and worry about how much I am learning and not worry about my grade or how “smart” I am perceived.”
Tenet 6 – Viewing others’ success as inspiration	2	“I learned to let the success of others motivate and inspire me rather than make me insecure.”
Tenet 7 – Believing in the malleability of intelligence	0	

The evaluation of this strengths‑based program demonstrates that introducing growth mindset concepts early in medical school is feasible and valuable. Quantitative evaluations from four cohorts show strong engagement, with nearly 90% of students agreeing that the large‑group session met its learning objectives. This consistent endorsement suggests that first‑year students respond positively to structured opportunities that link personal strengths with strategies for approaching medical training. Qualitative analysis reveals unprompted student familiarity with growth mindset principles. Over one‑third of students described ways they planned to apply growth mindset concepts to their first‑year experience, most commonly referencing the tenets “Believing effort yields improvement” and “Persisting through setbacks.” These tenets were likely referenced more as they are relevant to challenges faced during the transition to medical school, such as managing academic pressure, learning from critical feedback, and coping with uncertainty. Likewise, students wrote about learning from failure and embracing challenging opportunities. Fewer comments reflected tenets about inspiration from peers’ success or prioritizing learning over approval which may be due to the individual, classroom-based experiences they have had so far; these tenets may emerge more prominently as students encounter collaborative and evaluative environments in clinical training.

This study is limited by its single‑institution design and short‑term outcomes. Future work should examine whether growth mindset training influences long‑term well‑being and professional development. Nonetheless, these findings suggest that integrating strengths‑based growth mindset interventions early in the curriculum appears to provide a helpful framework for students to connect their individual strengths to growth‑oriented learning behaviors. Medical schools that want to implement this curriculum can consider using our seminar outline and discussion prompts (see Supplementary Material 1). Exposure to these principles may support resilience, adaptability, and reflection, all elements of success in medical education.

## Supplementary Information

Below is the link to the electronic supplementary material.

Supplementary Material 1
