# Supplementary material for: A New Approach to Cultivating Growth Mindset: Strengths-Based Training for First-Year Medical Students
Source: Med Sci Educ. 2026 Jun 19;36(4):1907–9. doi: 10.1007/s40670-026-02786-5 (PMC13582677; doi:10.1007/s40670-026-02786-5)
Supplement: Supplementary file 1 [file 40670_2026_2786_MOESM1_ESM.docx]

**Robert Wood Johnson Medical School Seminar on Leadership Strengths and Growth Mindset**

**Large Group Seminar:**

*This session was a seminar for the entire first year class which included a lecture on the following topics as well as opportunities for discussion with peers.*

- Overview of professional development and leadership in the healthcare setting
- Overview of how strengths can be harnessed for leadership and growth
- Explanation of the *CliftonStrengths®* assessment and how to interpret results
- Discussion of growth mindset vs. fixed mindset
- Impact of growth mindset on leadership

**Small Group Discussion:**

*In smaller groups (11-12 students per small group with a physician facilitator), students discussed the following prompts.*

Part One - Strengths of the Individual:

- Discuss the following questions:
  - Are you surprised about what came up as a Strength for you?
  - In what ways have you used your top talents in a past volunteer or leadership experience?
  - How might the application of these talents help you in navigating some of the challenges associated with medical school and practice?
  - Are there areas where your talents are turned up too high and get in the way of engaging meaningfully with others?

Part Two - Strengths of the Group/Team (Facilitators created a grid for students within the team to list each of their top 5 strengths):

- Open the grid depicting individual strengths of the team and discuss the following:
  - What story does this grid tell us regarding the diversity of strengths and talents of the group?
  - Looking at their distribution, how might we work well together? Where might we expect gaps?
  - When were you part of a successful team and what combination of strengths made that work?  When were you part of a team that did not work so well together and what might have gone wrong there? Knowing your own strengths and those of others, what might you do differently?
  - How do you see it playing out in medical school – like when working in anatomy lab, study groups or group presentations?
